# Monitoring Off-Shore Fishing in the Northern Indian Ocean Based on Satellite Automatic Identification System and Remote Sensing Data

**DOI:** 10.3390/s24030781

**Published:** 2024-01-25

**Authors:** Jie Li, Qianguo Xing, Xuerong Li, Maham Arif, Jinghu Li

**Affiliations:** 1 CAS Key Laboratory of Coastal Environmental Processes and Ecological Remediation, Yantai Institute of Coastal Zone Research, Chinese Academy of Sciences, Yantai 264003, China; lijie185@mails.ucas.ac.cn (J.L.); xrli@yic.ac.cn (X.L.); jinghuli@yic.ac.cn (J.L.); 2 Shandong Key Laboratory of Coastal Environmental Processes, Yantai 264003, China; 3 University of Chinese Academy of Sciences, Beijing 100049, China

**Keywords:** automatic identification system (AIS), fishing vessels, spatial–temporal distribution, northern Indian Ocean, sea surface temperature (SST), chlorophyll a concentration (Chl-a)

## Abstract

Satellite-derived Sea Surface Temperature (SST) and sea-surface Chlorophyll a concentration (Chl-a), along with Automatic Identification System (AIS) data of fishing vessels, were used in the examination of the correlation between fishing operations and oceanographic factors within the northern Indian Ocean from March 2020 to February 2023. Frequency analysis and the empirical cumulative distribution function (ECDF) were used to calculate the optimum ranges of two oceanographic factors for fishing operations. The results revealed a substantial influence of the northeast and southwest monsoons significantly impacting fishing operations in the northern Indian Ocean, with extensive and active operations during the period from October to March and a notable reduction from April to September. Spatially, fishing vessels were mainly concentrated between 20° N and 6° S, extending from west of 90° E to the eastern coast of Africa. Observable seasonal variations in the distribution of fishing vessels were observed in the central and southeastern Arabian Sea, along with its adjacent high sea of the Indian Ocean. Concerning the marine environment, it was observed that during the northeast monsoon, the suitable SST contributed to high CPUEs in fishing operation areas. Fishing vessels were widely distributed in the areas with both mid-range and low-range Chl-a concentrations, with a small part distributed in high-concentration areas. Moreover, the monthly numbers of fishing vessels showed seasonal fluctuations between March 2020 and February 2023, displaying a periodic pattern with an overall increasing trend. The total number of fishing vessels decreased due to the impact of the COVID-19 pandemic in 2020, but this was followed by a gradual recovery in the subsequent two years. For fishing operations in the northern Indian Ocean, the optimum ranges for SST and Chl-a concentration were 27.96 to 29.47 °C and 0.03 to 1.81 mg/m^3^, respectively. The preliminary findings of this study revealed the spatial–temporal distribution characteristics of fishing vessels in the northern Indian Ocean and the suitable ranges of SST and Chl-a concentration for fishing operations. These results can serve as theoretical references for the production and resource management of off-shore fishing operations in the northern Indian Ocean.

## 1. Introduction

The ocean serves as the vital blue granary, offering humanity abundant animal proteins and providing economic and employment-related social benefits [1]. Marine fisheries play a crucial role in the marine economy; understanding the changes in fishery resources could promote the economic growth of coastal countries. Currently, with the continuous increase in fishing vessels and progressive fishing technology, understanding the distribution ranges, fishing intensity and other information about fishing operations can indirectly reveal the changes in fishery resources. This information could provide necessary decision-making support for marine spatial planning and ecological conservation.

The Indian Ocean fishing ground is one of the world’s distant water operational fishing grounds [2,3], holding vast potential for fishery resources. The tropical continental shelf waters of the Indian Ocean are the most important fishing areas, hosting a variety of fish species with high reproductive rates, short reproductive cycles, and significant variations in catch quantities [4,5,6]. For instance, the Arabian Sea is home to abundant species such as the Scombridae and Clupeidae [7], while the coastal areas of the Bay of Bengal host a large population of catfish species [8]. According to the latest statistics from the Food and Agriculture Organization of the United Nations (FAO), the catch in the Indian Ocean reached 1.22 × 10^7^ tons in 2020 [9]. In this context, it is necessary to understand the distributions of fishery resources in the Indian Ocean. Studying the spatial–temporal distributions and variations of Indian Ocean fishing vessels through vessel monitoring could indirectly reach this goal.

Fishing logbooks and Vessel Monitoring System (VMS) are traditional methods for monitoring fishing vessels, providing effective ways to analyze the spatial–temporal distribution characteristics of fishing vessels by recording information such as navigation time, locations, and status. However, both of these methods have their limitations: fishing logbooks need to be brought back only after the vessels return to ports, and these logbooks often suffer from issues such as incomplete information and irregular entries because of the long periods of off-shore operations, leading to shortcomings in the accuracy and effectiveness [10]; VMS data also have limitations, including a low time resolution and restricted access [11,12]. Consequently, the accuracy and timeliness of the two kinds of data are insufficient, and there are certain defects in the analysis of the spatial–temporal distributions and change characteristics of fishing vessels by using these data.

The collection and use of real-time fishing data have provided robust support in recent years by technological advancements such as big data. Automatic Identification System (AIS) is extensively employed for real-time monitoring and identification of fishing vessels. It transmits information such as latitude, longitude, heading, and speed, thereby serving as a valuable data source for the monitoring and management of marine fishing operations. The development of satellite AIS further expands AIS applications in the prediction and analysis of the distribution of fishery resources and the location of central fishing grounds [13]. Ocean fishing vessels transmit data to ground stations through satellites, facilitating rapid information feedback with high data accuracy. This mechanism serves to effectively compensate for limitations in fishing logbooks and VMS data [14,15], enabling a better way to complete the characteristics mining of fishing operations.

AIS is a digital device and navigation system that utilizes network, communication, and electronic information display technology. It has been widely applied in the maritime field. With the significantly increasing installation rate of AIS devices on fishing vessels, research on fishery-related applications based on AIS data has been widely conducted. Researchers worldwide have achieved a series of achievements in fishing behavior identifications [16,17,18], fishing operation areas extractions [19], fishing intensity measurements [20,21], and ecological pressure assessment from fishing operations [22,23]. Therefore, AIS data could provide an effective way to study the spatial–temporal distribution and variation characteristics of fishing vessels in the Indian Ocean.

Compared with the southern waters, there are many coastal countries in the northern Indian Ocean, resulting in more complex traffic conditions. In this regard, this study leverages AIS data spanning from March 2020 to February 2023 to investigate the spatial–temporal distribution and variation characteristics of fishing vessels in the northern Indian Ocean, indirectly revealing the spatial–temporal distribution of fishery resources and providing information and technical support for marine ecological protection and marine spatial planning and management in the Indian Ocean.

## 2. Data and Methods

### 2.1. Study Area and Data

**Figure 1:**
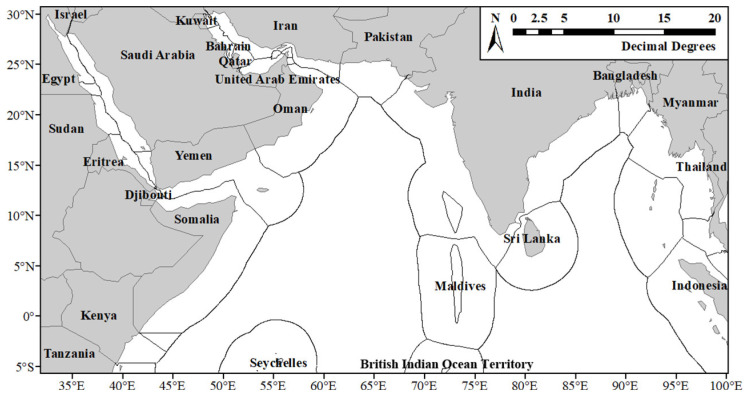
The northern Indian Ocean.

The study area covers the northern Indian Ocean from 32° E to 100° E and 31° N to 6° S, including the Red Sea, the Gulf of Aden, the Persian Gulf, the Gulf of Oman, the Arabian Sea, the Lakshadweep Sea, the Bay of Bengal, and the Andaman Sea. The coastal countries and their corresponding Exclusive Economic Zones (EEZs) are illustrated in Figure 1. This area is influenced by the Indian Ocean monsoon, featuring the southwest monsoon prevailing from June to September and the northeast monsoon from December to March of the next year. April to May and October to November are transition periods for the monsoons [24]. The monsoon transition induces alterations in ocean currents, subsequently impacting upwelling, seawater temperature, salinity, dissolved oxygen, and the distribution of plankton and other marine organisms [25]. Consequently, these changes impact the spatial distribution of fishery resources.

Data regarding the locations of fishing vessels were acquired from the MarineTraffic AIS data service website (https://www.marinetraffic.com, accessed on 1 March 2020 to 28 February 2023) for the period spanning from March 2020 to February 2023. The vessels’ positions were obtained through screenshots taken at 9:00 UTC, with a screenshot interval of one day. The AIS data service website marks the vessel positions with arrow-shaped symbols. In this study, a threshold method was used to extract these symbols, and the pixel coordinates of the centers of these symbols in the captured images were obtained. These pixel coordinates were then used to determine the vessel positions in the captured images. Leveraging the tile pyramid model [26], the pixel coordinates were converted to latitude and longitude coordinates to obtain the geographic positions of each fishing vessel. These coordinates were then imported into Matlab and ArcGIS software (Matlab R2021a and ArcMap 10.4.1) to analyze the spatial–temporal distribution of fishing vessels.

Sea Surface Temperature (SST) is a critical oceanographic component influencing the creation of fishing grounds since it directly affects the migration, reproduction, and spawning of marine organisms [27]. Chlorophyll a concentration (Chl-a) also plays a significant role in influencing the distribution of upper-layer fishery resources [28]. This study investigated the link between the marine environment and the spatial–temporal distribution of fishing vessels using data on SST and Chl-a concentration. SST and Chl-a concentration data were provided by SST and Chlor_a L3B data products from NASA’s Earthdata website (https://www.earthdata.nasa.gov, accessed on June 2020 to May 2023), which were obtained by the Moderate Resolution Imaging Spectrometer (MODIS) on board the Aqua satellite, with a temporal resolution of one month and a spatial resolution of 4 km. The data used in this study span the period from March 2020 to February 2023. The SST L3B data product is based on the method of the split-window nonlinear SST (NLSST) algorithm [29], and the Chlor_a L3B data product is based on the algorithm of Hu et al. [30], which combines the empirical band-difference method at low Chl-a concentrations and the ratio-transformation method at higher Chl-a concentrations.

### 2.2. Methods

#### 2.2.1. Characterization Indicators and Calculation Methods of the Spatial–Temporal Distribution Characteristics of Fishing Vessels

Based on the acquired AIS data, the interannual distribution heat of fishing vessels and the monthly and seasonal variations in fishing vessel numbers were selected as research indicators to analyze the spatial–temporal distribution of fishing vessels in the study area.

1. Normalized Heat Index

In order to depict the interannual spatial distribution of fishing vessels in the northern Indian Ocean from March 2020 to February 2023, this study presents interannual spatial distribution heat maps of fishing vessels in the study area. The map production process is as follows: First, the study area was divided into 10,064 (136 × 74) equally sized 0.5° × 0.5° grids. Using these 0.5° × 0.5° grids as the statistical unit, the annual numbers of fishing vessels in each grid were calculated. Then, the Normalized Heat Index (NHI) was obtained by normalizing the vessel numbers in each grid [31].

The NHI is used as the indicator to create the interannual spatial distribution heat maps of fishing vessels in the study area. The calculation formula of NHI is as follows:

(1) $$NHI_{i,j}=\frac{N_{i,j}-\mathrm{Min}}{\mathrm{Max}-\mathrm{Min}}$$

where *NHI_i_*_,*j*_ is the NHI for grid *j* in year *i*, *N_i_*_,*j*_ is the number of fishing vessels in grid *j* in year *i*, and Max and Min are the maximum and minimum values of fishing vessels numbers across all 0.5° × 0.5° grids for the three years, respectively. The range of *NHI_i_*_,*j*_ is from 0 to 1, with a higher *NHI_i_*_,*j*_ approaching 1 indicating a ‘hot’ grid in year *i*, reflecting more frequent fishing operations in the corresponding waters. Conversely, a lower *NHI_i_*_,*j*_ approaching 0 indicates a ‘cold’ grid, indicating fewer fishing operations.

2. Measurement method of time variations of fishing vessel numbers

The number of fishing vessels in a specific time period can also reflect the intensity of fishing operations. The greater number of fishing vessels presents stronger fishing operations. The statistical model for the number of fishing vessels is

(2) $$F=\sum_{i=1}^{n}F_{i}$$

where *F* is the number of fishing vessels in a specific time period, *F_i_* is the number of fishing vessels at moment *i*, and *n* represents time. For the convenience of analysis, this study calculated the number of fishing vessels with months and seasons as time units to measure the intensity of fishing operations in the northern Indian Ocean.

#### 2.2.2. Monthly Spatial Distribution Statistics of Fishing Vessels

In order to clarify the seasonal variation characteristics of fishing vessels’ spatial distribution in the study area, monthly AIS data were counted at a spatial resolution of 1° × 1°, and the monthly numbers of fishing vessels in each grid were obtained. To mitigate the impact of abnormal values and other factors, the medians of fishing vessel numbers in the same grid location and month from 2020 to 2023 were used as Catch Per Unit Effort (CPUE) in this grid [32]. Due to the presence of small numbers of fishing vessels in some grids, this study calculated the quartiles (Q1~Q3) of CPUE [33]. Using Q3 (the third quartile) as the threshold, grids with CPUEs higher than Q3 were defined as fishing operation areas in the northern Indian Ocean. These grids were visually represented and analyzed. From March 2020 to February 2023, there were 8456 grids with CPUEs higher than Q3, which were counted monthly in the 1° × 1° grids, with quartile values as follows: Q1 = 2, Q2 = 6, Q3 = 15.

#### 2.2.3. Oceanographic Factor Spatial Analysis

Monthly SST and Ch-a concentration data from March 2020 to February 2023 were also used in this study. The monthly median values of SST and Chl-a concentration were computed for the same month and the same latitude and longitude positions over the three years, and the monthly spatial distributions of SST and Chl-a concentration of the study area were obtained. Subsequently, these distributions were overlaid with fishing operation areas, and the monthly spatial distribution maps for fishing operation areas with SST and Chl-a concentration were created, which could facilitate the qualitative analysis of the relationship between fishing operations and oceanographic factors.

Finally, the quantitative analysis was conducted by extracting the corresponding oceanographic factor values based on the latitude and longitude coordinates of the fishing vessels. The variation ranges of oceanographic factors in fishing operation areas were identified and the Analysis of Variance (ANOVA) was used to compare the extracted data.

#### 2.2.4. Oceanographic Factor Preferred Range Calculation

Frequency analysis and the empirical cumulative distribution function (ECDF) [34,35,36] were used to calculate the preferred ranges of two oceanographic factors (SST and Chl-a concentration) for fishing operations in the northern Indian Ocean.

Firstly, the means, standard deviations, and preferred ranges (means ± standard deviations) of SST and Chl-a concentration for fishing operation areas in the study area were computed. Subsequently, the association between the oceanographic factors and CPUEs during the same period was analyzed by ECDF, and the CPUE-weighted preferred ranges of SST and Chl-a concentration were computed (factor values at the maximum *D*(*t*) ± standard deviations). The intersection of the two ranges represents the optimum SST and Chl-a concentration for fishing operations in the study area. In this analysis, three functions were used as follows:

(3) $$\begin{array}{l} F(t)=\frac{1}{n}\sum_{i=1}^{n}l(x_{i}),\;l(x_{i})=\left\{ \begin{array}{l} 1,\;x_{i}\leq t\\ 0,\;x_{i}>t \end{array}\right.\\ G(t)=\frac{1}{n}\sum_{i=1}^{n}\frac{y_{i}}{\bar{y}}l(x_{i})\\ D(t)=\left|F(t)-G(t)\right| \end{array}$$

where *F*(*t*) is the empirical cumulative distribution function, *G*(*t*) is the CPUE-weighted cumulative distribution function, *l*(*x_i_*) is the indication function, and *D*(*t*) is the absolute value of the difference between the two curves *F*(*t*) and *G*(*t*) at any point *t*. *n* is the number of fishing vessels, *x_i_* is the oceanographic factor of the fishing vessel *i*, *t* is the index ranking the ordered observations from the lowest to highest values of the oceanographic factors, *y_i_* is the CPUE of the fishing vessel *i*, and $\bar{y}$ is the mean of CPUE for all fishing vessels.

In the above work, the calculation and statistics were completed using Matlab R2021a software, and the heat maps and monthly spatial distribution maps were produced with ArcMap 10.4.1 software.

## 3. Results

### 3.1. Statistics of Fishing Vessels

**Figure 2:**
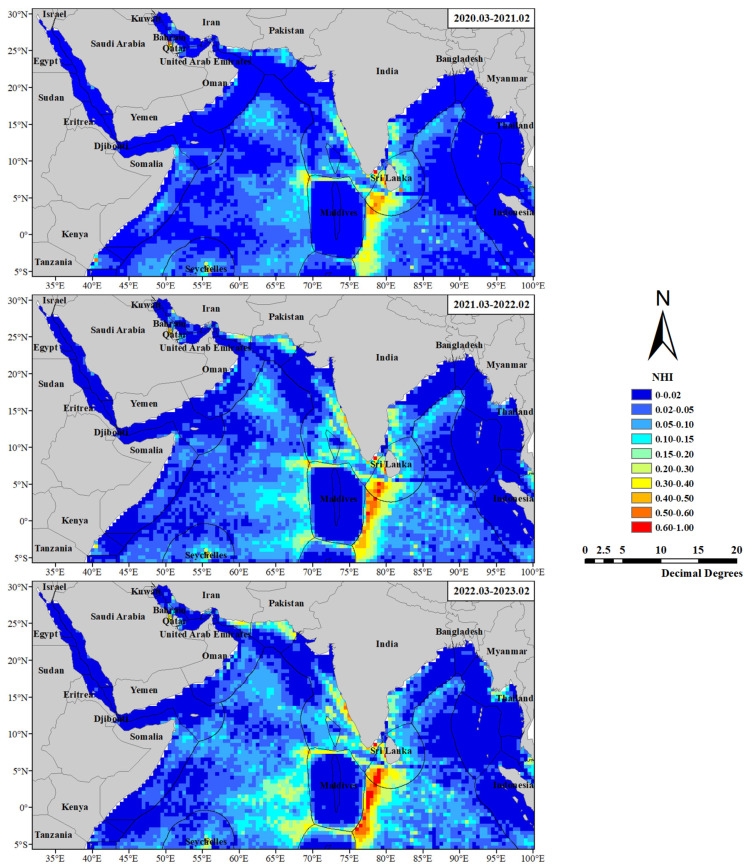
Interannual distribution heat maps of fishing vessels.

Figure 2 shows the interannual distribution heat maps of fishing vessels in the northern Indian Ocean. It is evident that fishing vessels are highly concentrated in the surrounding areas of the EEZ of the Maldives, the Palk Strait, the coastal areas of India, the Seychelles, and the Gulf of Bahrain. Fishing vessels are mostly active in fishing grounds and aquaculture areas, presenting an overall clustered distribution. Over the three years, the heat of fishing vessels increased annually. In addition to the mentioned regions, the heat of fishing vessels gradually increased in the coastal areas of Iran, Pakistan, Myanmar, Thailand, and the central Arabian Sea. There are also areas with consistently low fishing vessel heat in the study area, such as the Red Sea, the Gulf of Aden, the northern and southeastern areas of the Bay of Bengal, the Andaman Sea, the eastern Indian Ocean, the EEZ of the Maldives, and the British Indian Ocean Territory. Furthermore, in the center of the Bay of Bengal, fishing vessels exhibit a strip-like distribution, with heat increasing first and then decreasing.

**Figure 3:**
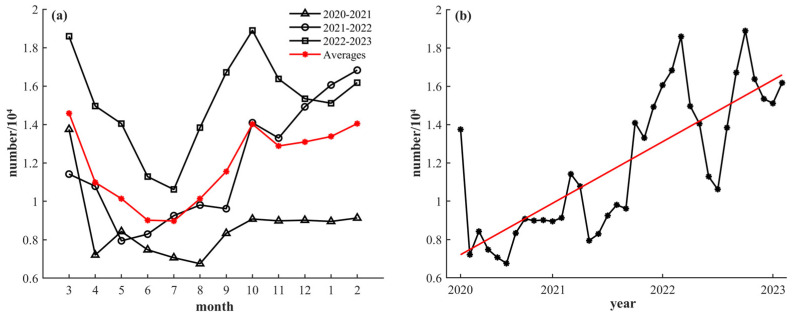
Monthly variations and trend analysis of the numbers of fishing vessels. (**a**) Monthly variations. (**b**) Trend analysis. The averages and trend lines are shown in red.

Figure 3a shows the monthly variation lines chart of the total fishing vessels in the study area from 2020 to 2023. In three years, the number of fishing vessels drastically declines after March and then rises around September. Throughout the period from October to March of the next year, the numbers of fishing vessels remain relatively high, with March and October exhibiting counts higher than their adjacent months (February and April, September and November, respectively).

The monthly numbers of fishing vessels exhibit periodic variations from March 2020 to February 2023, and the trend analysis of the total numbers of fishing vessels increases markedly (Figure 3b, where the trend line is represented in red, and the relevant parameters are shown in Table 1, *p* < 0.05).

According to the averages of fishing vessel numbers in different seasons and the relative variation rates of the annual averages in the study area, the fishing vessel numbers have obvious seasonal differences, showing the characteristics of winter > autumn > spring > summer (Table 2). Over the three years, the number of fishing vessels from March 2020 to February 2021 is lower than the following two years, with a negative relative variation rate in the spring of 2021 (fishing season).

### 3.2. Monthly Spatial Distribution Characteristics of Fishing Vessels

**Figure 4:**
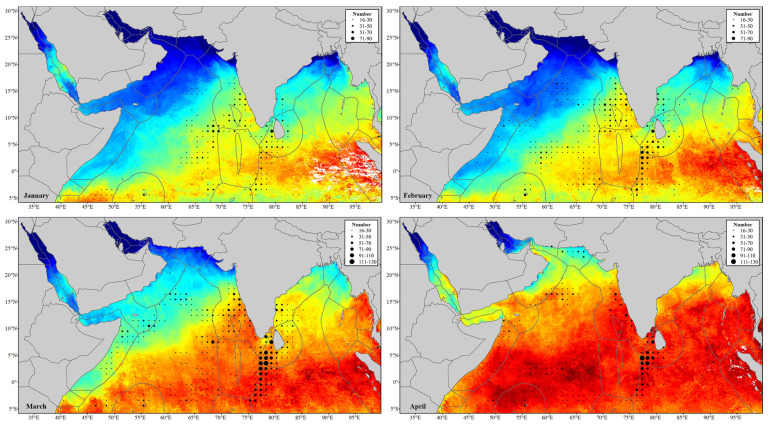

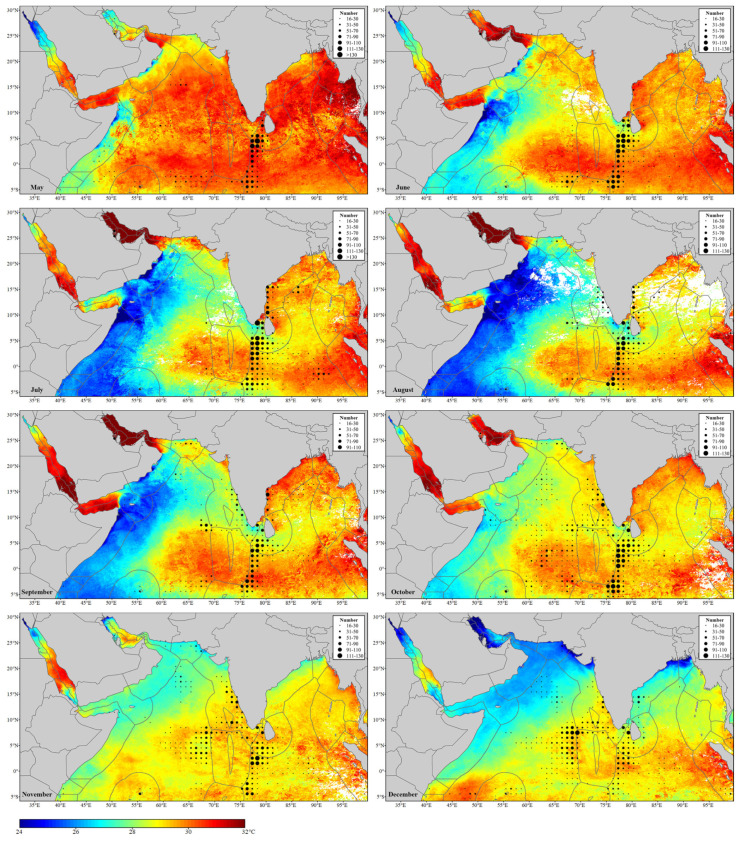
Monthly spatial distribution of fishing vessels and SST.

Figure 4 shows the monthly distribution of fishing operation areas in the northern Indian Ocean. It is evident that the latitudinal positions of fishing operation areas (CPUE > Q3) mainly concentrate between 20° N and 6° S, and the longitudinal positions are mainly located in the west of 90° E to the eastern coast of Africa. There are also some scattered fishing operation areas outside this range. During the northeast monsoon period (October to March), there are more fishing operation areas located in the mentioned range, while during the southwest monsoon period (April to September), fishing operation areas are fewer and scattered.

CPUEs in the Gulf of Aden, the Persian Gulf, the Gulf of Oman, and the northern Arabian Sea are generally low and there is no CPUE over Q3 in the Red Sea all through the year. High CPUEs are observed in the Bay of Bengal during both the northeast monsoon (December to March) and southwest monsoon (June to September) periods.

From October to March, fishing vessels exhibit a broad distribution range, extending from the central Arabian Sea to the west of the EEZ of the Maldives. The CPUE remains relatively consistent during the period from October to December, showing an increase compared to the months from January to March. The distribution range of fishing vessels narrows from April to May, leading to a decline in CPUE. CPUE reaches its minimum in June to July and gradually rises from August to September. This area exhibits clear seasonal variations in the distribution of fishing vessels, making it the most significant area for variations in fishing vessels distribution within the study area.

Fishing operation areas are observed in the EEZ of Somalia from February to June and from September to November. These areas are mainly concentrated in the south of Ras Hafun from February to June, with increasing CPUEs from February to March and decreasing from April to June. Fishing operation areas are mostly focused in the waters between Alula and Bayla (Somalia) from September to November, with a higher CPUE in October.

In the southwestern waters of Sri Lanka to 6° S in the Indian Ocean, CPUE remains consistently high allthrough the year. From June to December, an increase in fishing operation areas is noted in the eastern Indian Ocean, but CPUE is relatively low. From January to May, there is generally no fishing operation in this waters.

### 3.3. Fishing Operations with SST

The monthly spatial distributions of SST are also shown in Figure 4. It is observed that in the waters where fishing vessels are concentrated (20° N~6° S), there is a large warm water area extending from the eastern of the Indian Ocean to the eastern coast of Africa from January to March, leading to an increase in CPUE. During April and May, SST reaches its peak (mostly over 29 °C), leading to a subsequent decrease in CPUE. From June to August, the high SST area (over 29 °C) contracts, contributing to a further decrease in CPUE. SST rises in October relative to September as the southwest monsoon down, which leads to a rise in CPUE. By November, SST becomes relatively uniform, contributing to a decrease in CPUE. In December, the high-SST area shifts southward, leading to an increase in CPUE in the western area of the EEZ of the Maldives.

From June to September, the eastern coast of Africa is influenced by cold currents, resulting in a lower SST (24 to 27 °C) compared to other months. In this period, the low SST area shows a distinct band-shaped area and without fishing operation area.

The SST ranges of the fishing operations areas in each season were calculated, also with the means and variances. The ANOVA was used to test the seasonal effects. All results are shown in Table 3.

It can be seen that over the three years, the average SST ranges of the fishing operation areas in the four seasons are as follows: 20.44 to 33.98 °C in spring, 24.76 to 35.77 °C in summer, 24.45 to 33.73 °C in autumn, and 18.38 to 32.22 °C in winter. The means show the characteristic order of spring > summer > autumn > winter.

In the variance analysis of each year, the Mean Squares Between (MSB) are much higher than the Mean Squares Within (MSW). At the significance level of α = 0.05, the *F*-values are also much higher than the critical value (*F*_crit_ = 2.60), indicating a significant seasonal impact on the SST of the fishing operation areas.

### 3.4. Fishing Operations with Chl-a Concentration

**Figure 5:**
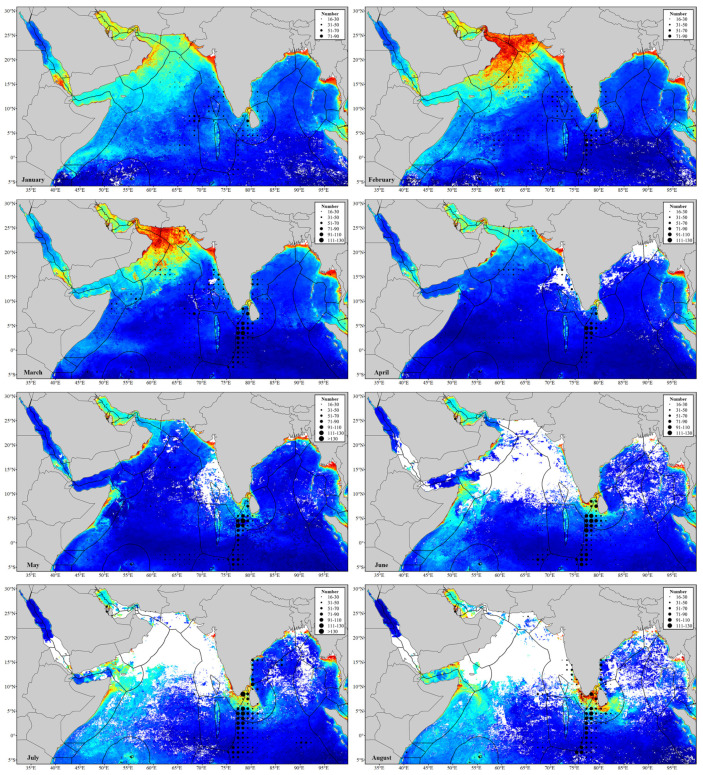

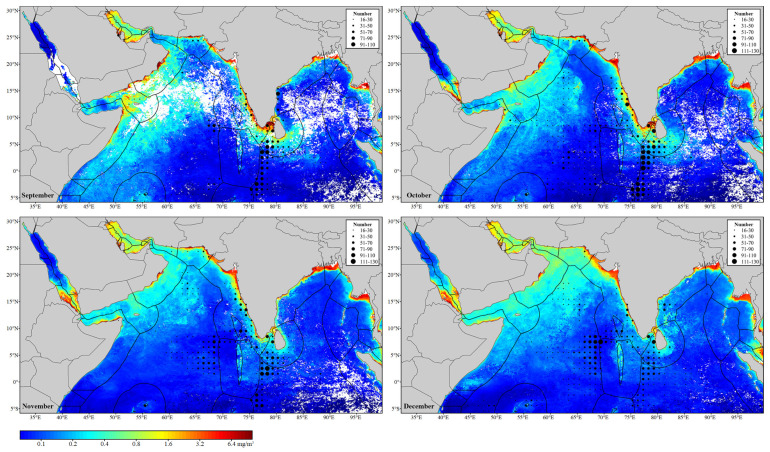
Monthly spatial distribution of fishing vessels and Chl-a concentration.

Monthly spatial distributions of fishing vessels and Chl-a concentration are shown in Figure 5. It is observed that, under the influence of the monsoon, Chl-a concentration in the Arabian Sea and the eastern coast of Africa varies significantly, whereas other areas exhibit relatively stable conditions.

In the Arabian Sea, the high Chl-a concentration area generally exhibits a latitudinal pattern during the northeast monsoon period. It extends southward from the northern Arabian Sea in December and contracts northward in March. Fishing vessels exhibit movements northward and southward in response to changes in the latitudinal position of areas with high Chl-a concentration and are mainly distributed between the high- and low-concentration areas.

In the eastern coast of Africa, Chl-a concentration gradually increases from south to north during the southwest monsoon period. The high concentration area could extend to the central Arabian Sea. Conversely, during the northeast monsoon period, Chl-a concentration increases from north to south, with the high-concentration area extending to the coast of Kenya. From February to June, fishing vessels in the south of Ras Hafun are concentrated in both mid-value and low-value areas of Chl-a concentration. From September to November, fishing vessels in the waters from Alula to Bayla are concentrated in the mid-value area of Chl-a concentration.

Table 4 shows the Chl-a concentration ranges means, variances, and variance analysis results of fishing operations areas in each season. Over the three years, the average Chl-a concentration ranges of the fishing operation areas for each season are as follows: 0.05 to 19.79 mg/m^3^ in spring, 0.05 to 69.10 mg/m^3^ in summer, 0.04 to 87.89 mg/m^3^ in autumn, and 0.05 to 23.70 mg/m^3^ in winter. The means in summer and autumn are higher than those in spring and winter.

In the variance analysis of each year, the MSB is also much higher than the MSW. At the significance level of α = 0.05, the *F*-values are also much higher than the critical value (*F*_crit_ = 2.60). Compared with SST, the Chl-a concentration of the fishing operation areas with lower MSB and higher MSW result in lower *F*-values than SST. This indicates that the Chl-a concentration of the fishing operation areas in the study area is less affected by seasonal variations compared with SST.

### 3.5. Oceanographic Factor Preferred Ranges

**Figure 6:**
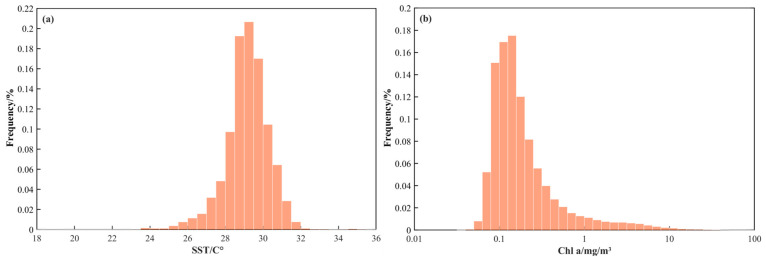
Frequency distribution histograms of SST/Chl-a concentration with CPUE. (**a**) SST. (**b**) Chl-a concentration.

The ranges of SST and Chl-a concentration for fishing operations in the northern Indian Ocean are 18.09 to 35.80 °C and 0.03 to 96.54 mg/m^3^, respectively (Figure 6). The frequency distribution histograms of SST/Chl-a concentration with CPUE exhibit a skewed distribution trend. The mean of SST is 29.15 °C and the standard deviation is 1.19, while the mean of Chl-a concentration is 0.46 mg/m^3^ with the standard deviation of 1.73. By frequency analysis, approximately 74.70% of fishing vessels are concentrated in the area where SST ranges from 27.96 to 30.34 °C (29.15 ± 1.19 °C), and 96.21% are concentrated in the area where Chl-a concentration ranges from 0.03 to 2.19 mg/m^3^ (0.46 ± 1.73 mg/m^3^).

**Figure 7:**
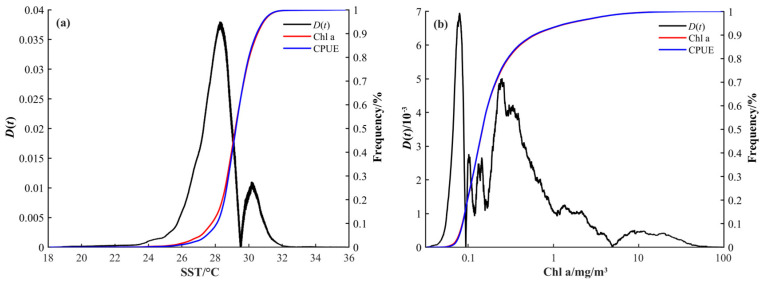
Empirical cumulative distribution function for CPUE and SST (**a**), Chl-a concentration (**b**).

The ECDF analysis results are shown in Figure 7. The maximum *D*(*t*) values corresponding to SST and Chl-a concentration occur at 28.28 °C and 0.08 mg/m^3^, respectively, different from their respective means (29.15 °C and 0.46 mg/m^3^). Then, the CPUE-weighted preferred ranges of SST and Chl-a concentration are obtained: 27.09 to 29.47 °C (28.28 ± 1.19 °C) and 0.03 to 1.81 mg/m^3^ (0.08 ± 1.73 mg/m^3^), respectively.

Combining the results of frequency analysis and ECDF, intersecting two preferred ranges, the optimum ranges of SST and Chl-a concentration for fishing operations in the northern Indian Ocean are determined as 27.96 to 29.47 °C and 0.03 to 1.81 mg/m^3^.

## 4. Discussion

### 4.1. The Numbers and Spatial–Temporal Distribution Variations of Fishing Vessels

The winter northeast monsoon and summer southwest monsoon have significant impacts on the distribution of fishing vessels in the northern Indian Ocean [37]. The northeast monsoon periodis characterized by weaker wind (mostly at grade 3 to 4), dry and clear weather, and good visibility on the sea surface, making this period suitable for fishing operations. On the other hand, during the summer monsoon period, the wind gets stronger (reaching up to grade 6 to 7 in the Arabian Sea and up to grade 10 in its western area), also with humid and rainy weather and decreased visibility, making this period unsuitable for fishing operations [38]. Additionally, the spawning period for many catch targets occurs in summer. The summer southwest monsoon induces upwellings, transferring nutrients from the lower layers to the surface waters and creating favorable conditions for the reproduction of the catch targets. The reduced fishing operations during this period are beneficial for minimizing disturbances to the breeding activities and promoting the growth and development of the catch targets [39]. Therefore, the northeast monsoon period (October to March) is often the fishing season, with widespread distribution and frequent fishing operations, while the southwest monsoon period (April to September) is considered the fishing moratorium, with few fishing vessels distributed.

In the southwest monsoon period, a majority of fishing vessels typically return. The distribution of fishing vessels decreases in the Arabian Sea along with its adjacent high sea of the Indian Ocean, while a few vessels navigate to areas less affected by the southwest monsoon in the eastern Indian Ocean for fishing operations, such as the Bay of Bengal and its adjacent waters [40]. This shift could contribute to high CPUEs in this area compared with the period from November to March (fishing season). As the southwest monsoon gradually subsides in September, fishing vessels start returning to the operation areas. Consequently, the distribution of fishing vessels in this month appears in a strip-like pattern, as seen in the Bay of Bengal and the eastern Indian Ocean. In October, as the fishing season commences and some fishing vessels are still in the process of relocating, the CPUE of the south waters of India reaches its peak for the entire year.

The high fishing vessel numbers in March and October compared with their adjacent months (February and April, September and November, respectively) are likely related to the end and beginning of the fishing season. Furthermore, a rise in fishing vessels in March could be related to the warmer weather [41]. Due to the impact of the COVID-19 pandemic [42,43,44,45], the number of fishing vessels was relatively low in 2020 (Table 2). As countries gradually lifted pandemic control measures in 2021 [46], fishing operations resumed and the number of fishing vessels began to recover, which also led to a significant increase during the fishing season (December 2021 to February 2022). The monthly numbers of fishing vessels showed an upward trend from 2020 to 2023 (Figure 3). The underlying reasons for this trend, aside from the impact of the COVID-19 pandemic, also include national fishery management policies [5], the abundance of fishery resources [47], and the upgrading of the data acquisition website. Nevertheless, specific reasons behind this trend necessitate further research for a comprehensive understanding.

### 4.2. The Impacts of Oceanographic Factors on Fishing Operations

According to the CPUE spatial–temporal distribution maps (Figure 4 and Figure 5), there is a significant difference in CPUE between the northeast monsoon and the southwest monsoon period. CPUE increases during the northeast monsoon, while it decreases during the southwest monsoon. CPUE is closely related to the spawning and migration of fishery resources [48] and is also influenced by the seasonal variations due to monsoons. Seasonal variations in SST and Chl-a concentration caused by the monsoons eventually affect the distribution of fishing grounds.

SST is a key oceanographic factor affecting the distribution of fishing grounds, which could indicate the distribution and variation of fishing grounds. From April to May, in the fishing operation areas between 20° N and 6° S, SST is mostly above 29 °C due to continuous sunlight exposure. From June to September, the combined effects of the southwest monsoon, typhoons, surface water mixing, evaporation, and surface wind stress reduce the heat of sea surface water, leading to a decrease in SST [49]. This period also witnesses the influence of the Somali Current, resulting in the appearance of a low-SST area (24 to 27 °C) along the east coast of Africa and the western Arabian Sea. CPUE is relatively low in these months, suggesting that, aside from the impacts of the fishing moratorium and monsoon, excessively high or low SSTs can also contribute to a decrease in CPUE. From October to March, SST in the area between 20° N and 6° S is mostly concentrated in the range of 26 to 30 °C. Therefore, during the southwest monsoon period, the SST in fishing operation areas varies significantly, whereas during the northeast monsoon period, the SST remains relatively stable. The analysis indicates that the suitable SST during the northeast monsoon period is identified as an environmental factor contributing to high CPUE in the fishing operation areas.

The optimum SST range for fishing operations in the northern Indian Ocean obtained in this study is 27.96 to 29.47 °C. This range is generally lower than the optimum SST range of 29.3 to 30.8 °C reported by Xu et al. [50]. The difference in results could be attributed to the different study years. Xu et al.’s study took place during an El Niño year (2016), which led to an increase in SST [51]. The dynamic nature of oceanographic conditions underscores the importance of considering temporal variations in assessing the optimum SST for fishing operations.

Chl-a concentration, representing the content of bait and plankton, is also used for predicting fishing ground locations [52]. Seasonal variations in Chl-a concentration are seen in the Arabian Sea and the eastern coast of Africa. Chl-a concentration is generally high all through the year in the northern Arabian Sea, especially in the Gulf of Oman and its adjacent waters [53]. In the southern Arabian Sea, Chl-a concentration decreases, while in the central waters, it remains in an intermediate range between high and low values. This distribution pattern could be a contributing factor to the distribution of fishing operation areas in the central Arabian Sea. In the eastern coast of Africa, fishing operation areas are concentrated in the areas with both mid-range and low-range Chl-a concentrations during the northeast monsoon period. However, from June to September, there is a low distribution of fishing vessels in this area. Aside from the impacts of the southwest monsoon and fishing moratorium, the low-temperature and high-Chl-a concentration area formed by cold currents and upwellings may inhibit fishery resources, leading to a decrease in CPUE. Additionally, there are fishing operation areas in coastal waters with high Chl-a concentration, such as the coastal areas of Pakistan and Cape Comorin (India). In summary, the impact of Chl-a concentration on fishing operations in the study area lacks a standardized criterion, which indicates that the relationship between fishing operations and Chl-a concentration is not straightforward. In order to understand the relationship comprehensively, more detailed fishery resource data, such as the biological characteristics of the catch targets, are necessary for future research.

The optimum Chl-a concentration range for fishing operations in the northern Indian Ocean obtained in this study is 0.03 to 1.81 mg/m^3^, which differs from the optimum range of 0.2 to 0.5 mg/m^3^ reported by Yanget al. [54]. The difference in results could be attributed to differences in the study areas, considering coastal areas or not.

### 4.3. Speculations on the Locations and Seasonal Variations of Fishing Grounds in the Northern Indian Ocean

Based on the spatial–temporal distribution of fishing vessels in the study area, in addition to coastal waters and EEZs, there are also fishing grounds in high seas. Preliminary inferences suggest the presence of fishing grounds in the central water of the Arabian Sea (20°~11° N, 58°~70° E), the eastern and western waters of the EEZ of the Maldives (9° N~5° S, 60°~70° E; 6° N~5° S, 77°~85° E), the high sea of the Bay of Bengal (18°~12° N, 83°~90° E), and the northeastern Indian Ocean (1° N~3° S, 88°~94° E). Among these areas, fishing vessels are distributed throughout the year in the eastern waters of the Maldives’ EEZ, while in other areas, fishing vessel distributions change with seasons, which are related to factors such as geographical location, monsoon, and the different breeding, feeding, and migration periods of different catch targets. For instance, in the central water of the Arabian Sea, there are abundant catches of catch targets such as Indian mackerels (*Rastrelliger kanagurta*) and sardines. These species spawn in coastal waters during the southwest monsoon period and migrate to off-shore waters for feeding during the northeast monsoon period [55,56], which leads to fishing vessels in this water mostly operating during the northeast monsoon period. The eastern waters of theMaldives’ EEZ are abundant in tuna, including various species that spawn and migrate in different seasons. Some species, such as bigeye tuna (*Thunnus obesus*) and yellowfin tuna (*Thunnus albacares*), can be caught throughout the year [57]. This diversity contributes to fishing operations in this area throughout the year.

The above speculations need to be further analyzed, combined with the biological characteristics of the catch targets. Understanding the migratory habits of the catch targets will also help reveal the fishing ground changes in high seas.

### 4.4. AIS Data Issues

This study collected AIS data through website screenshots, providing only location information. In comparison with original AIS data, the obtained data have limitations, including lack of information, incomplete display of vessel points, and low resolution due to website permissions and display classification. In future research, we intend to improve data acquisition by using original AIS data, which contain more detailed information about fishing vessels, including vessel types, status, and specific operation times.

## 5. Conclusions

This study investigated spatial–temporal distribution characteristics of fishing vessels in the northern Indian Ocean from March 2020 to February 2023 by using AIS data. Combined with simultaneous SST and Chl-a concentration data, spatial overlay maps of fishing operation areas and oceanographic factors were created. Additionally, monthly and interannual variations in the number of fishing vessels were analyzed. The following conclusions are drawn: (1) The northeast monsoon and southwest monsoon have significant impacts on the distribution of fishing vessels in the northern Indian Ocean. Fishing season occurs during the northeast monsoon period (October to March), while the transition and fishing moratorium take place during the southwest monsoon period (April to September). (2) Fishing vessels are mainly concentrated between 20° N and 6° S, extending from west of 90° E to the eastern coast of Africa. (3) The suitable SST contributed to high CPUEs in fishing operation areas during the northeast monsoon and fishing vessels are widely distributed in the areas with both mid-range and low-range Chl-a concentrations. (4) The monthly numbers of fishing vessels show seasonal variations, displaying a periodic pattern with an overall increasing trend. Due to the impact of the COVID-19 pandemic, the total number of fishing vessels decreased in 2020, but this was followed by a gradual recovery in the subsequent two years. (5) The optimum ranges of SST and Chl-a concentration for fishing operations are defined as 27.96 to 29.47 °C for SST and 0.03 to 1.81 mg/m^3^ for Chl-a concentration.

The application of AIS data can describe the characteristics of fishing vessel operations in detail, which could help fishery authorities understand the spatial–temporal distribution of fishing operations and improve or reasonably formulate fishery management policies. However, given the lack of catch data and the deficiency of collected data, this study still has limitations. In future research, there is an intention to explore the driving factors behind the spatial–temporal distribution of fishing vessels and identify key factors influencing this distribution with original AIS data and catch data.

## Figures and Tables

**Table 1:** Linear regression of monthly variations of the numbers of fishing vessels.

Slope	Intercept	*R* ^2^
268.09	6941.11	0.60

**Table 2:** Seasonal variation statistics of the numbers of fishing vessels.

Time	Year	Spring (March to May)		Summer (June to August)		Autumn (September to November)		Winter (December to February)	
	Average/Month	Average/Month	Relative Variation Rate/%	Average/Month	Relative Variation Rate/%	Average/Month	Relative Variation Rate/%	Average/Month	Relative Variation Rate/%
03.2020–02.2021	8681	9794	12.82	7098	−18.24	8799	1.36	9034	4.07
03.2021–02.2022	11,859	10,049	−15.26	9117	−23.12	12,334	4.01	15,935	34.37
03.2022–02.2023	15,163	15,865	4.63	11,914	−21.43	17,326	14.26	15,545	2.52
03.2020–02.2023	11,901	11,903	0.02	9376	−21.22	12,820	7.72	13,505	13.48

**Table 3:** Statistics and variance analysis of SST of fishing operation areas in each season.

Year	Season	Min (°C)	Max (°C)	Mean (°C)	Var	MSB	MSW	*F*
03.2020–02.2021	Spring	20.26	34.56	29.96	1.74	5268.02	1.42	3708.29
	Summer	24.56	35.80	29.75	1.59			
	Autumn	25.56	34.41	29.33	0.74			
	Winter	18.41	32.93	28.64	1.49			
03.2021–02.2022	Spring	20.68	33.76	29.81	1.44	5549.84	1.17	4741.54
	Summer	25.04	35.75	29.22	0.93			
	Autumn	23.62	33.24	29.10	0.69			
	Winter	18.10	32.50	28.63	1.50			
03.2022–02.2023	Spring	20.37	33.62	29.72	1.57	8684.58	1.07	8105.29
	Summer	24.68	35.77	29.14	1.13			
	Autumn	24.17	33.54	28.81	0.54			
	Winter	18.62	31.23	28.38	1.25			

**Table 4:** Statistics and variance analysis of Chl-a concentration of fishing operation areas in each season.

Year	Season	Min (mg/m^3^)	Max (mg/m^3^)	Mean (mg/m^3^)	Var	MSB	MSW	*F*
03.2020–02.2021	Spring	0.04	20.30	0.26	0.62	1263.56	4.33	291.80
	Summer	0.05	73.04	0.86	13.98			
	Autumn	0.05	96.54	0.79	6.27			
	Winter	0.06	19.56	0.29	0.27			
03.2021–02.2022	Spring	0.05	12.19	0.20	0.17	1102.74	2.64	417.70
	Summer	0.06	50.36	0.48	2.53			
	Autumn	0.05	86.98	0.77	7.14			
	Winter	0.06	28.75	0.39	0.99			
03.2022–02.2023	Spring	0.05	26.89	0.38	1.11	285.41	2.60	109.91
	Summer	0.04	83.91	0.53	5.16			
	Autumn	0.03	80.16	0.50	3.16			
	Winter	0.03	22.79	0.29	0.48			

## Data Availability

The data presented in this study are available on request from the corresponding author. The data are not publicly available due to privacy protection reasons.
